# Use of a “critical difference” statistical criterion improves the predictive utility of the Health Assessment Questionnaire-Disability Index score in patients with rheumatoid arthritis

**DOI:** 10.1186/s41927-019-0095-2

**Published:** 2019-12-10

**Authors:** Frank Behrens, Michaela Koehm, Eva C. Schwaneck, Marc Schmalzing, Holger Gnann, Gerd Greger, Hans-Peter Tony, Harald Burkhardt

**Affiliations:** 1 Division of Rheumatology, University Hospital Frankfurt, Goethe University, Frankfurt am Main, Germany; 2 Fraunhofer Institute for Molecular Biology and Applied Ecology IME, Project Group Translational Medicine & Pharmacology TMP, Frankfurt am Main, Germany; 3 Schwerpunkt Rheumatologie/Klinische Immunologie Medizinische Klinik und Poliklinik II, Universität Würzburg, Würzburg, Germany; 4 Abteilung Biostatistik, GKM Gesellschaft für Therapieforschung mbH, Munich, Germany; 5 AbbVie Deutschland GmbH & Co. KG, Wiesbaden, Germany

**Keywords:** Health Assessment Questionnaire, Functional ability, Rheumatoid arthritis, Adalimumab

## Abstract

**Background:**

The Health Assessment Questionnaire-Disability Index (HAQ-DI) is used to assess functional status in rheumatoid arthritis (RA), but the change required for meaningful improvements remains unclear. A minimum clinically important difference (MCID) of 0.22 is frequently used in RA trials. The aim of this study was to determine a statistically defined critical difference for HAQ-DI (HAQ-DI-d_crit_) and evaluate its association with therapeutic outcomes.

**Methods:**

We retrospectively analyzed data from adult German patients with RA enrolled in a multicenter observational trial in which they received adalimumab therapy at the decision of the treating clinician during routine clinical care. The HAQ-DI-d_crit_, defined as the minimum change that can be reliably discriminated from random long-term variations in patients on stable therapy, was determined by evaluating intra-individual variation in patient scores. Other outcomes of interest included Disease Activity Score-28 joints and patient-reported pain and fatigue.

**Results:**

The HAQ-DI-d_crit_ was calculated as an improvement (decrease) from baseline of 0.68 in a discovery cohort (*N* = 1645) of RA patients on stable therapy and with moderate disease activity (mean DAS28 [standard deviation] of 4.4 [1.6]). In the full patient cohort (*N* = 2740), 22.1% of patients achieved a HAQ-DI-d_crit_ improvement at month 6. Compared with patients with a small improvement in HAQ-DI (decrease of ≥0.22 to < 0.68) or no improvement (< 0.22), patients achieving a HAQ-DI-d_crit_ at month 6 had better therapeutic outcomes at months 12 and 24, including stable functional improvements. Change in pain was the most important predictor of HAQ-DI improvement during the first 6 months of therapy.

**Conclusions:**

A HAQ-DI-d_crit_ of 0.68 is a reliable measure of functional improvement. This measure may be useful in routine clinical care and clinical trials.

**Trial registration:**

ClinicalTrials.gov NCT01076205. Registered on February 26, 2010 (retrospectively registered).

## Background

The Health Assessment Questionnaire-Disability Index (HAQ-DI) is considered the gold standard for the assessment of function in patients with rheumatoid arthritis (RA) [1] and is the clinical variable most closely associated with joint replacement, work disability, and mortality [2]. This tool is scored on a scale of 0 (minimum disability) to 3 (maximum disability) and encompasses eight domains of daily living [3]. As a stand-alone measure, the HAQ-DI is frequently used as a primary or secondary endpoint in randomized controlled trials in patients with RA [4], and it is routinely incorporated into the American College of Rheumatology (ACR) improvement criteria as an option for functional assessment [5].

Despite the importance of this tool in measuring physical function, the level of HAQ-DI change required for a clinically important and robust improvement in an individual patient remains unclear. Several studies have evaluated the minimum clinically important difference (MCID) for the HAQ-DI using different methodologies and patient populations. An MCID of 0.22 was determined by Wells et al. on the basis of a single evening of conversations between 40 RA patients with differing functional status [6], and this value has been applied in randomized clinical trials of therapeutic agents [7, 8]. On the higher end of the scale, Wolfe et al. determined a “really important difference” of 0.87 in 8931 RA patients aged < 65 years based on subjective measures of functional independence, and a difference of 0.74 based on objective reports of work disability [9]. Other studies have found intermediate values [10–13]. In addition to the wide variation in HAQ-DI MCIDs, some experts have criticized the methodology involved in calculating MCIDs from patient-reported outcomes such as the HAQ-DI based on ordinal measures in which distances between each raw score point are unequal; conversion to interval scaling based on Rasch model-transformed scales has been recommended as an alternative [14].

We have developed a statistical method for determining thresholds for individual therapeutic responses based on the magnitude of change required to exceed random variation during long-term stable therapy, termed the “critical difference” (d_crit_) [15, 16]. This approach was piloted using the Disease Activity Score-28 joints (DAS28); achievement of the DAS28-d_crit_, a DAS28 decrease (improvement) of ≥1.8 from baseline, was shown to be a stable and robust indicator of a positive individual therapeutic response in patients with active RA initiating adalimumab therapy [15]. A later study successfully applied this same method to patient-reported outcomes, including pain and fatigue [16].

The observational studies on which the previous reports were based used the Funktionsfragebogen Hannover patient questionnaire as the functional assessment. A subsequent observational study used the HAQ-DI as a measure of self-reported function, thereby allowing us to apply the critical difference methodology to this important assessment. The aim of this study was to determine a statistically defined critical difference for HAQ-DI (HAQ-DI-d_crit_) and evaluate its association with therapeutic outcomes. Our data suggest that this criterion would be useful both in the evaluation of individual patients during routine clinical care and as a response criterion in randomized clinical trials.

## Patients and methods

### Study design

This study used data from German patients with RA enrolled in a multicenter observational trial who received adalimumab therapy at the decision of the treating clinician during routine clinical care (Clinicaltrials.gov NCT01076205). Adult patients (≥18 years of age) were required to have a diagnosis of active RA, a clinical indication for treatment with a tumor necrosis factor inhibitor, and no contraindications. Patients included in these analyses were treated between January 12, 2009, and September 14, 2017. All patients were informed of the objectives of the observational study and gave written consent for their voluntary participation in the study and the anonymous use of personal data in statistical analyses. Ethics approval was obtained from the Ethics Commission of the Medical Department of Goethe University, Frankfurt am Main, Germany (No. 122/09).

The discovery cohort, which was used to determine the HAQ-DI-d_crit_, included only patients who were on stable therapy (no change in adalimumab dose or concomitant therapies) from month 12 to 24 and had HAQ-DI data for the month 12 and 24 visits. The requirement for stable treatment allowed intra-individual fluctuations in outcomes to be distinguished from responses due to alterations in therapy. No other exclusion criteria were applied.

For the full cohort analyses, patients were required to have baseline data for DAS28 and HAQ-DI and month 6 data for HAQ-DI. Patients who were previously treated with adalimumab, were in functional remission (HAQ-DI ≤ 0.5), or had low disease activity (DAS28 ≤ 3.2) at baseline were excluded from these analyses. All patients who met the specified criteria were included in the full cohort analyses.

### Outcomes

The analyses reported here include data up to 24 months. Visits were conducted at baseline (month 0, prior to initiation of adalimumab therapy) and months 3, 6, 12, and 24. Disease activity was assessed by DAS28 [17] and function was assessed by HAQ-DI [3]; for both measures, higher scores indicate greater impairment. At each visit, patients provided self-assessments of pain, fatigue, and global health in the past 7 days on an 11-point categorical scale ranging from 0 (best) to 10 (worst).

### Statistical analyses

Statistical analyses were performed with SAS® statistical software (Version 9.4). Summary statistics are presented for demographic and disease characteristics. Missing data were not imputed. Patient numbers varied at different visits because of study discontinuations and missing data for specified outcomes.

The method for determining the HAQ-DI statistically defined critical difference (HAQ-DI-d_crit_), the minimum change that can be reliably discriminated from random variations in patients on stable therapy, was based on evaluations of intra-individual variation in patients undergoing stable therapy (discovery cohort) between month 12 and month 24 as described previously [15, 16]. These evaluations allowed us to determine the long-term reliability of the HAQ-DI over a period of months, rather than its short-term measurement error. Long-term variation is more applicable to real-life patient care in which assessment of disease activity is usually performed at intervals separated by several months. Briefly, we adapted the method of Lienert and Raatz [18] to determine a critical difference based on the one-sided 5% z-value of the normal distribution in patients on stable therapy from months 12 to 24 after initiation of adalimumab [19]. A one-sided critical difference was calculated because only improvements (decreases) in HAQ-DI were relevant to defining a response. Pearson correlation and the standard deviation were used to determine the standard error of measurement for the HAQ-DI-d_crit_. The HAQ-DI-d_crit_ value was then used to evaluate functional improvement in the full cohort of patients initiating adalimumab therapy. Stepwise multiple regression analysis incorporating 29 variables, including demographic characteristics, comorbidities, concomitant treatment, and measures of disease activity, was used to identify predictors for improvement in HAQ-DI at month 6.

## Results

### Determination of the critical difference in HAQ-DI

The discovery cohort consisted of 1645 patients who were on stable therapy from month 12 to month 24 after initiation of adalimumab. Seventy-two percent were female, and mean baseline values (SD) were disease duration of 10.9 (9.0) years, DAS28 of 4.4 (1.6), and HAQ-DI of 1.1 (0.72). The HAQ-DI-d_crit_ value in this discovery cohort was determined to be 0.641. Subgroup analyses by baseline characteristics showed that HAQ-DI-d_crit_ values ranged from a low of 0.597 for patients with baseline HAQ-DI < 1 to a high of 0.673 for patients with baseline HAQ-DI ≥ 1 (Table 1). On the basis of this subgroup analysis, we chose a HAQ-DI-d_crit_ value of 0.68 as a conservative value representing a statistically valid individual improvement in HAQ-DI score that exceeded the threshold of random fluctuation.

**Table 1:** Determination of HAQ-DI-d_crit_ in patients on stable therapy

Population	N	Standard deviation	Pearson correlation coefficient	Standard error measurement	One-sided HAQ-DI-d_crit_
Total discovery cohort	1645	0.73	0.86	0.39	0.641
Men	453	0.66	0.85	0.37	0.604
Women	1186	0.75	0.86	0.40	0.655
Aged < 60 years at baseline	986	0.68	0.83	0.39	0.646
Aged ≥60 years at baseline	652	0.76	0.87	0.39	0.634
Baseline HAQ-DI < 1	684	0.44	0.66	0.36	0.597
Baseline HAQ-DI ≥1	914	0.70	0.83	0.41	0.673

*Abbreviations: HAQ-DI Health Assessment Questionnaire-Disability Index, HAQ-DI-d*_*crit*_ *critical difference for change beyond random variation in the HAQ-DI (decrease ≥ 0.68 from baseline)*

In the full cohort of patients initiating treatment with adalimumab (all patients who met inclusion/exclusion criteria for the analysis), 522 of 2740 patients (19.1%) achieved a HAQ-DI-d_crit_ improvement (HAQ-DI decrease ≥0.68 from baseline) at 3 months. The HAQ-DI-d_crit_ achievement rates increased slightly during the study to 639 of 2895 (22.1%) at month 6, 544 of 2193 (24.8%) at month 12, and 443 of 1532 (28.9%) at month 24.

### Characteristics of patients by improvement in HAQ-DI at 6 months

The statistically determined HAQ-DI-d_crit_ of 0.68 was higher than many other values used to assess a HAQ-DI response, including the MCID value of 0.22 sometimes used in clinical trials [7, 8]. We therefore decided to compare characteristics and outcomes in patient subgroups on the basis of achievement of various HAQ-DI criteria at month 6, the visit at which many specialists make the decision to continue or modify therapy. We could not directly compare patients with a HAQ-DI decrease ≥0.68 with those achieving a decrease ≥0.22 because the latter, less stringent target also included all patients in the HAQ-DI-d_crit_ group. We therefore categorized patients in the full patient cohort (*N* = 2895 at 6 months) into the following 3 subgroups based on change in HAQ-DI at 6 months: (1) patients achieving a HAQ-DI-d_crit_ improvement (decrease ≥0.68), (2) patients achieving an MCID of 0.22 but less than the HAQ-DI-d_crit_ (HAQ-DI decrease of ≥0.22 to < 0.68; referred to as “small improvement”), and (3) patients with no or minimal HAQ-DI improvement (HAQ-DI decrease < 0.22; referred to as “no improvement”). Because these groups were biased by the functional criteria used to define them, statistical differences between them were not assessed; Table 2 provides descriptive data only.

**Table 2:** Patient baseline characteristics by change in HAQ-DI between month 0 and month 6

Baseline characteristic	HAQ-DI-d_crit_ improvement (≥0.68)	Small improvement (≥0.22 to < 0.68)	No improvement (< 0.22)
n	639	961	1295
Age, years	53.2 (13.4)	56.2 (12.4)	57.7 (11.7)
Women, %	72.1	73.5	78.6
BMI, kg/m^2^	26.3 (4.9)	27.1 (5.6)	27.8 (6.1)
Disease duration, years	7.5 (7.2)	9.7 (8.6)	10.4 (9.7)
DAS28	5.4 (1.2)	5.3 (1.2)	5.1 (1.1)
HAQ-DI	1.60 (0.51)	1.48 (0.56)	1.44 (0.55)
Pain^a^	6.9 (1.9)	6.4 (2.0)	6.2 (2.1)
Fatigue^a^	6.5 (2.4)	6.0 (2.5)	5.9 (2.5)
Patient global assessment^a^	6.8 (2.1)	6.3 (2.0)	6.1 (2.1)
Tender joint count	9.2 (6.8)	9.7 (7.0)	8.9 (6.8)
Swollen joint count	6.8 (5.8)	6.7 (5.8)	5.7 (5.2)

*Abbreviations: BMI* body mass index, *DAS28* Disease Activity Score-28 joints, *HAQ-DI* Health Assessment Questionnaire-Disability Index, *HAQ-DI-d*_*crit*_ critical difference for change beyond random variation in the HAQ-DI (decrease ≥0.68 from baseline)

Data are presented as mean (standard deviation) unless otherwise indicated; complete data were not available for all patients

^a^Measured on a categorical scale ranging from 0 (best) to 10 (worst)

Greater improvements in HAQ-DI at month 6 were more common in younger patients and those with a lower body mass index (BMI) and shorter disease duration (Table 2). The three subgroups had generally comparable DAS28 scores at baseline, although the group with no HAQ-DI improvement had the lowest disease activity. A similar pattern was seen with baseline HAQ-DI values: the group with the greatest HAQ-DI improvement at month 6 had the highest mean baseline HAQ-DI values and the group with no improvement had the lowest.

### Association of HAQ-DI change criteria with other outcomes

To explore the predictive value of different levels of HAQ-DI change at month 6 with respect to additional therapeutic response outcomes, such as DAS28, we evaluated outcomes in patients in each of the 3 subgroups at months 12 and 24. During the first 24 months of the observational study, 31.2% of patients withdrew, most commonly because of a lack of effectiveness, and 18.9% were lost to follow-up. As might be expected from responder bias, study withdrawal rates were higher in the subgroup with no HAQ-DI improvement (28% at month 12 and 37% at month 24) than in the group with a small HAQ-DI improvement (18.7% at month 12 and 27.3% at month 24) or HAQ-DI-d_crit_ improvement (15.3% at month 12 and 25.2% at month 24).

Patients who achieved a HAQ-DI-d_crit_ improvement at month 6 consistently showed better outcomes at months 12 and 24 than patients with lower levels of HAQ-DI improvement (Table 3). Differences in outcomes were observed in both mean values and response criteria, including DAS28 remission and DAS28-d_crit_ response (DAS28 improvement ≥1.8 from baseline). For instance, in patients who achieved a HAQ-DI-d_crit_ response at month 6, the rate of DAS28 remission at month 12 was approximately 20% higher than in patients with a small HAQ-DI improvement and approximately 30% higher than patients with no HAQ-DI improvement (DAS28 remission rates of 46.6, 25.2, and 17.5%, respectively).

**Table 3:** Patient outcomes by change in HAQ-DI between month 0 and month 6

	HAQ-DI-d_crit_ improvement (≥0.68) at month 6 (*n* = 639)		Small improvement (≥0.22 to < 0.68) at month 6 (*n* = 961)		No improvement (< 0.22) at month 6 (*n* = 1295)	
Outcome	Month 12	Month 24	Month 12	Month 24	Month 12	Month 24
n	536	385	782	553	928	638
*Mean values (standard deviation)*						
DAS28	2.90 (1.26)	2.86 (1.25)	3.48 (1.33)	3.30 (1.38)	3.83 (1.31)	3.62 (1.27)
Pain^a^	3.1 (2.4)	3.1 (2.5)	4.1 (2.4)	3.8 (2.4)	4.9 (2.3)	4.5 (2.3)
Fatigue^a^	3.3 (2.6)	3.4 (2.6)	4.2 (2.6)	3.8 (2.7)	4.9 (2.6)	4.5 (2.6)
PGA^a^	3.1 (2.3)	3.3 (2.5)	4.2 (2.3)	3.8 (2.3)	4.9 (2.3)	4.5 (2.2)
Tender joint count	1.9 (3.3)	1.9 (3.3)	3.6 (4.9)	3.3 (5.0)	4.5 (5.9)	3.8 (5.3)
Swollen joint count	1.3 (2.3)	1.2 (2.5)	2.3 (3.6)	1.9 (3.3)	2.5 (4.0)	2.0 (3.5)
*% of patients*						
HAQ-DI remission (≤0.5)	52.2	54.2	24.7	26.2	8.71	11.1
DAS28 remission (≤2.6)	46.6	48.7	25.2	34.8	17.5	24.4
DAS28-d_crit_ response (≥1.8 from baseline)	66.0	68.4	50.1	53.5	32.1	36.2
Pain-d_crit_ response (≥3 from baseline)	67.5	67.9	45.2	47.6	27.2	31.3
Fatigue-d_crit_ response (≥3 from baseline)	59.7	51.9	38.2	42.5	23.4	27.9
PGA-d_crit_ response (≥3 from baseline)	67.5	63.9	43.7	48.7	28.7	31.4

*Abbreviations: DAS28* Disease Activity Score-28 joints, *d*_*crit*_ critical difference for change beyond random variation, *HAQ-DI* Health Assessment Questionnaire-Disability Index, *PGA* patient global assessment

Complete data were not available for all patients

^a^Measured on a categorical scale ranging from 0 (best) to 10 (worst)

### Stability of HAQ-DI changes during therapy

**Fig. 1:**
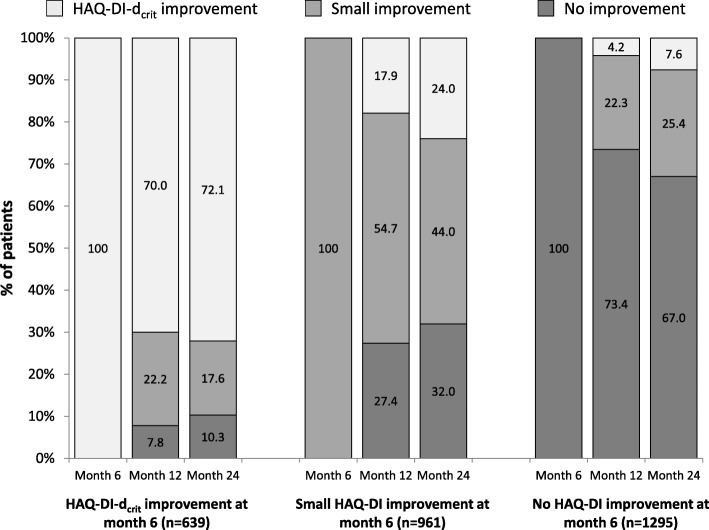
Stability of HAQ-DI changes during therapy. Continued achievement of HAQ-DI improvement criteria at months 12 and 24 was evaluated in patient subgroups based on HAQ-DI improvement at month 6. HAQ-DI-d_crit_ improvement was defined as HAQ-DI change from baseline ≥0.68, small improvement as ≥0.22 to < 0.68, and no improvement as < 0.22. Differences in patient numbers from Table 3 are due to the absence of HAQ-DI data in some patients. *HAQ-DI* Health Assessment Questionnaire-Disability Index, *HAQ-DI-d*_*crit*_ critical difference for change beyond random variation in the HAQ-DI (decrease ≥0.68 from baseline)

The stability of a therapeutic response in patients remaining on therapy reflects both the continued efficacy of the treatment and the consistency of the response tool. To evaluate the stability of the HAQ-DI-d_crit_ response, we assessed the proportions of patients with a HAQ-DI-d_crit_ response at month 6 who maintained this response at subsequent visits during continued adalimumab therapy. Approximately 70% of patients with a HAQ-DI-d_crit_ response at month 6 also had a HAQ-DI-d_crit_ response at months 12 and 24 (Fig. 1). Most patients who did not sustain the HAQ-DI-d_crit_ response moved into the small improvement category (HAQ-DI decrease from baseline of ≥0.22 to < 0.68). Patients with no improvement also had stable responses; about 70% had no improvement at both subsequent time points. In contrast, only about half of the patients with a small HAQ-DI improvement at month 6 maintained this level of improvement at months 12 (54.7%) and 24 (44.0%). The remaining patients in this subgroup were fairly equally distributed between a HAQ-DI-d_crit_ improvement (about 20%) and no improvement (about 30%).

### Predictors for change in HAQ-DI from month 0 to month 6

A stepwise multiple regression model was used to identify predictors of HAQ-DI improvement during the first 6 months of adalimumab therapy (Table 4). The most important predictor was change in pain, as assessed by a patient-reported 11-point categorical pain scale, between month 0 and month 6; greater improvement in pain was associated with greater improvement in HAQ-DI. A high baseline HAQ-DI score was a positive predictor for improvement in HAQ-DI, but a high baseline pain score was a negative predictor. Other negative predictors included older age, longer disease duration, higher BMI, and higher baseline DAS28. As with pain, greater improvement in DAS28 from month 0 to month 6 was associated with greater improvement in HAQ-DI during this time period.

**Table 4:** Stepwise regression model for predictors of change in HAQ-DI from month 6 to month 0 (*P* < 0.001)

Variable	Coefficient^a,b^	Partial R^2^	Cumulated R^2^
Change in pain (month 6 – month 0)^c^	0.10333	0.27893	0.2789
Baseline HAQ-DI	−0.35821	0.05436	0.333
Baseline pain	0.06382	0.04669	0.3800
Age	0.00493	0.02033	0.4003
Change in DAS28 (month 6 – month 0)^c^	0.06488	0.01115	0.4115
Disease duration	0.00645	0.00813	0.4196
Body mass index	0.00730	0.00537	0.4250
Baseline DAS28	0.04143	0.00450	0.4295

Model intercept = −0.62463

*Abbreviations: DAS28* Disease Activity Score-28 joints, *HAQ-DI* Health Assessment Questionnaire-Disability Index

^a^The coefficient shows the influence for each unit of the predictor’s scale

^b^Because a negative value for HAQ-DI month 6 – month 0 indicates an improvement, variables with negative coefficients are positive predictors and those with positive coefficients are negative predictors

^c^Higher values on these scales represent greater impairment, so higher values for month 6 – month 0 correspond to lack of improvement and are a negative predictor for improvement in HAQ-DI

## Discussion

The HAQ-DI is a validated assessment of function that effectively discriminates active treatment from placebo [20] and predicts key RA outcomes, including work disability and mortality [2]. It is frequently used in RA clinical trials, observational studies, and daily patient care, and is considered the gold standard measurement of function in rheumatology [1, 4]. Over the years, there have been many approaches to determining a clinically significant improvement in HAQ-DI. Many of these approaches have used anchor-based assessments involving either subjective (eg, patient’s view of their overall disease status) or objective (eg, documented work disability) measures [6, 9, 12, 13, 19]. Some analyses were based on population-based means [8], whereas others were based on between-patient differences [6, 10, 12]. HAQ-DI MCIDs range widely in value depending on the specific study and there is concern about the accuracy of calculations based on an ordinal rather than interval scale [14].

Our approach to determining a valid criterion for HAQ-DI improvement is different from previous efforts: our goal was to establish a change in HAQ-DI that exceeded long-term random fluctuation within an individual patient on stable therapy. Long-term changes encompass short-term measurement variability as well as nonsystematic changes in disease activity during stable therapy. The short-term test-retest reliability of the HAQ-DI is quite high, as indicated by an intraclass correlation of 0.897 (95% confidence interval, 0.855–0.927) for two assessments taken 1 to 2 days apart [21]. However, patients in rheumatology clinical care are typically seen at 3- to 6-month intervals, so long-term variability is more relevant to outcomes observed during clinical care.

We found that the degree of change required to exceed normal long-term variation in a discovery cohort (*N* = 1645) on stable therapy with moderate disease activity and a mean disease duration of 10.9 years was a HAQ-DI improvement (decrease) of ≥0.68 points. Of the various MCIDs previously reported, the d_crit_ value is closest to the 0.74 “really important difference” determined from objective reports of work disability [9]. In the full patient cohort (*N* = 2740), 22.1% achieved a HAQ-DI-d_crit_ response at month 6 after initiation of adalimumab therapy. Approximately 70% of patients who achieved a HAQ-DI-d_crit_ response at month 6 retained it at months 12 and 24. The stability of the HAQ-DI-d_crit_ criterion over 18 months is especially noteworthy given that disease-related deterioration in function occurs over time in patients with RA [22]. In contrast, patients in the small improvement subgroup showed considerable variation in HAQ-DI responses at subsequent time points, with some improving and some deteriorating.

Our observation that achievement of a HAQ-DI MCID of 0.22 is in some cases due to random variation, rather than an improvement in function, is in keeping with a previous study by Wolfe et al. involving 50 patients with RA followed over approximately 16 years [23]. This study found that the HAQ-DI within-patient variation between assessments (approximately one per year) was 0.436, only slightly below the between-patient variation of 0.596, and almost twice as large as an MCID of 0.22. It is likely that the extensive within-patient variation contributes to the high rates of HAQ-DI MCID achievement observed in some clinical trials. In one recent study, 43% of patients in the placebo arm of a randomized trial achieved a HAQ-DI MCID of 0.22 at 3 months (prior to being switched to active treatment) [7].

An examination of baseline patient characteristics based on the magnitude of HAQ-DI change at month 6 showed that the subgroup achieving a HAQ-DI-d_crit_ improvement at month 6 had a lower mean age, lower BMI, and shorter disease duration than patients in the subgroups with a small HAQ-DI improvement (between the frequently used MCID of 0.22 and 0.68) or no improvement (< 0.22). Baseline mean HAQ-DI scores were somewhat higher in the HAQ-DI-d_crit_ subgroup than in the other subgroups, perhaps because responder criteria are easier to achieve with high baseline disease activity [24].

Because the derivation of the HAQ-DI-d_crit_ was based on statistical parameters and not on patient-centered anchors, it was critical to evaluate whether a HAQ-DI-d_crit_ response was associated with clinically relevant outcomes. We found that patients achieving a HAQ-DI-d_crit_ response at month 6 not only had higher rates of HAQ-DI remission at months 6 and 12, but also markedly higher rates of DAS28 remission and therapeutic responses for DAS28, pain, fatigue, and patient global health than patients in the other subgroups. Similarly, mean values for the objective assessments of tender and swollen joint counts were lower in the group achieving a HAQ-DI-d_crit_ response. It is perhaps not surprising that a more stringent functional response criterion is associated with better function at later time points. However, the association between the HAQ-DI-d_crit_ criterion and other outcomes, such as DAS28 remission and improvement in patient-reported outcomes, indicates that HAQ-DI-d_crit_ functional improvements are linked to meaningful differences in subsequent patient clinical status compared with the small improvement and no improvement groups.

Using a stepwise regression model, we identified change in pain from month 0 to month 6 as the most important predictor of change in HAQ-DI during the first 6 months of adalimumab therapy; this variable accounted for > 25% of the HAQ-DI change variance observed in this model. High baseline pain was a negative predictor for HAQ-DI improvement. Other studies concur on the impact of pain on function [16, 23, 25, 26]. Pain has been identified as the largest component of HAQ-DI [23] and an explanatory variable for all subdimensions of this functional assessment tool [26]. In addition to being correlated with function, pain is also strongly associated with DAS28; 68% of patients achieving a DAS28 therapeutic response, as assessed by the DAS28-d_crit_, also achieved a significant improvement in pain [16]. Together, these data suggest that pain is an important driver of therapeutic outcomes. We further identified high baseline HAQ-DI as a positive predictor for improvements in HAQ-DI from month 0 to month 6, likely due to the greater window for improvement in patients with high baseline scores. As others have observed, one of the most important drawbacks of HAQ-DI as a functional assessment is a floor effect in which patients with low baseline HAQ-DIs cannot experience significant HAQ-DI decreases despite clinical improvement [1].

This study has several important limitations. Although the HAQ-DI-d_crit_ was derived from a large sample size, the discovery cohort was limited to German patients preparing to initiate adalimumab therapy. Accordingly, patients with different ethnicities or milder or earlier disease may have a different HAQ-DI-d_crit_ limit than the one reported here. As our data indicate, the HAQ-DI-d_crit_ for patients with baseline HAQ-DI < 1 is 0.597, rather than the higher number we used as a conservative value in this study. It is therefore possible that the HAQ-DI-d_crit_ used in the study reported here is too high for patients with milder RA. We hope our statistical methods will be applied to varied groups of patients in other countries to provide insights into variations in HAQ-DI-d_crit_ values in different populations and with different disease severities. In addition, it is important to note that individual patients may experience meaningful benefits with HAQ-DI improvements lower than the statistically determined HAQ-DI-d_crit_. However, as we have shown in this study, on a population-wide basis lower HAQ-DI improvements may be due to random fluctuation and are unlikely to be as clinically relevant or as stable as a HAQ-DI-d_crit_ response. We acknowledge that patients who initiate treatment with good physical function are not well suited for this measure because of the fairly large change required to achieve a HAQ-DI-d_crit_ response; we excluded patients who were in functional remission (HAQ-DI < 0.5) from our analyses. As noted previously, floor effects (the inability of patients with low baseline HAQ-DIs to experience significant HAQ-DI decreases despite clinical improvement) are an issue with the HAQ-DI, and this tool is not appropriate for detecting change within the range of normal physical function [1].

## Conclusions

Our data indicate that the statistically determined HAQ-DI-d_crit_ value of 0.68 represents a robust change in function that can be distinguished from long-term random fluctuation. The clinical relevance of this measure is shown by the fact that achievement of a HAQ-DI-d_crit_ corresponds to other patient-reported and objective therapeutic outcomes. The stability of this criterion and its ability to reliably predict future functional status distinguishes it from other commonly used measures of HAQ-DI improvement that rely on smaller reductions. We hope our study will help extend the utility of HAQ-DI assessments in both randomized clinical trials and daily clinical practice.

## Data Availability

The datasets used in this study are available from the corresponding author on reasonable request.

## Abbreviations

ACR: American College of Rheumatology
BMI: Body mass index
DAS28: Disease Activity Score based on 28 joints
DAS28-d_crit_: Critical difference for change beyond random variation in the DAS28 (improvement of ≥1.8 from baseline)
HAQ-DI: Health Assessment Questionnaire-Disability Index
HAQ-DI-d_crit_: Critical difference for change beyond random variation in the HAQ-DI (decrease ≥0.68 from baseline)
MCID: Minimum clinically important difference
RA: Rheumatoid arthritis
